# Quantitative Electroencephalography and Behavioural Correlates of Daytime Sleepiness in Chronic Stroke

**DOI:** 10.1155/2014/794086

**Published:** 2014-05-06

**Authors:** Katherine Herron, Derk-Jan Dijk, Philip Dean, Ellen Seiss, Annette Sterr

**Affiliations:** ^1^Pain Management Centre, National Hospital for Neurology and Neurosurgery, University College London Hospitals, London WC1N3BG, UK; ^2^Surrey Sleep Research Centre, Faculty of Health and Medical Sciences, University of Surrey, Guildford GU2 7XH, UK; ^3^Department of Psychology, Faculty of Arts and Human Science, University of Surrey, Guildford GU2 7XH, UK

## Abstract

Sleepiness is common after stroke, but in contrast to its importance for rehabilitation, existing studies focus primarily on the acute state and often use subjective sleepiness measures only. We used quantitative electroencephalography (qEEG) to extract physiological sleepiness, as well as subjective reports, in response to motor-cognitive demand in stroke patients and controls. We hypothesised that (a) slowing of the EEG is chronically sustained after stroke; (b) increased power in lower frequencies and increased sleepiness are associated; and (c) sleepiness is modulated by motor-cognitive demand. QEEGs were recorded in 32 chronic stroke patients and 20 controls using a Karolinska Drowsiness Test protocol administered before and after a motor priming task. Subjective sleepiness was measured using the Karolinska Sleepiness Scale. The findings showed that power density was significantly increased in delta and theta frequency bands over both hemispheres in patients which were not associated with subjective sleepiness ratings. This effect was not observed in controls. The motor priming task induced differential hemispheric effects with greater increase in low-frequency bands and presumably compensatory increases in higher frequency bands. The results indicate sustained slowing in the qEEG in chronic stroke, but in contrast to healthy controls, these changes are not related to perceived sleepiness.

## 1. Introduction

Daytime sleepiness is commonly experienced in the acute phase of stroke [1, 2] which becomes a chronic problem in 34% of patients sustaining beyond six months [3–7]. Poststroke sleepiness is associated with lower quality of life [5], affects the ability to return to work [8], impacts cognitive functioning [9, 10], and hinders rehabilitation participation and outcome [7, 11–13]. Furthermore, the increased number of accidents committed by persons with a history of stroke has been attributed to sleepiness and fatigue [14, 15]. Several factors may contribute to sleepiness in stroke patients including neurological damage per se [16], depression [17], low social interaction [3], medication side effects [18–20], insomnia [21], sleep disordered breathing [17], and general poor health [5]. Strokes that affect the motor cortex may result in partial, or full, paralysis of the limbs. These limitations in motor functioning require increased effort for most activities of daily living, resulting in a greater level of exhaustion and sleepiness [22].

Sleepiness is complex construct to quantify and measure. Sleepiness describes an increased drive to sleep [23] which is distinct from fatigue. Fatigue refers to exhaustion as a result of physical or mental strain but this does not necessarily require sleep to be reversed [24]. Sleepiness can be measured subjectively using questionnaires; however, this only captures perceived sleepiness which can be influenced by other factors such as experimenter bias, mood, motivation, and poor introspection [25–27]. The latter has been particularly observed in those with brain injury and sleep problems [4, 17, 28]. Therefore, the objective measurement of physiological arousal is more accurate for detecting sleepiness levels. This requires the recording of neural activity in real time, and therefore, more sophisticated technology, that is, electroencephalography (EEG). Decreased physiological arousal, or sleepiness, is indicated by EEG frequency changes. Frequencies can be grouped into four distinct frequency bands: delta (1–3 Hz), theta (4–7 Hz), alpha (8–12 Hz), and beta (>12 Hz). The EEG in the normal waking adult consists primarily of beta and alpha rhythms. When arousal decreases, the rhythm becomes dominated by alpha and eventually lower frequencies when transitioning to sleep (>8 Hz). EEG methods have been incorporated into protocols for measuring sleepiness with a greater level of accuracy compared to questionnaire approaches alone.

A commonly used protocol is the Multiple Sleep Latency Test (MSLT) [29]. The MSLT aims to quantify sleepiness propensity by recording the EEG whilst the participant is given an opportunity to sleep in a laboratory. Sleep onset is determined by visual scoring of the EEG when the criteria for stage 1 sleep are met for 90 seconds [29]. A shorter sleep onset latency, measured in minutes, indicates higher the sleep propensity.

The MSLT protocol does not provide additional information about the characteristics of the EEG per se. But through offline analysis of the frequency composition of the raw EEG at a particular time point, the power in the slower frequencies associated with increased sleepiness (>12 Hz) can be determined to provide important indicators for how sleepy a person is whilst awake. This process is known as quantitative EEG (qEEG). Karolinska Drowsiness Test (KDT) [30] utilises qEEG to quantify sleepiness [31]. In the KDT protocol, the EEG is recorded whilst participants are awake and qEEG analyses are applied offline. Studies have shown that increased power in the lower frequency bands, mostly alpha and theta, correlates with subjective ratings of sleepiness across multiple time points in healthy adults [32–34]. Slowing of the waking EEG is generally recognised as a physiological indicator of subjectively experienced sleepiness, and indeed, increased sleep propensity [33].

Although sleepiness is often reported after stroke which brings additional problematic consequences, the majority of studies rely on subjective reports thus rendering our understanding of sleepiness in this population as limited which has implications for effective patient management. Objective neurophysiological methods, such as qEEQ [31–35], are lacking in this population but could help to improve the quantification of the level of severity of sleepiness problems in this population.

To the best of our knowledge, qEEG has not yet been used as a tool to investigate sleepiness in chronic stroke patients with chronic deficits. Several studies have used qEEG in acute stroke patients in the context of identifying neurological abnormalities and reported slowing in the delta and theta frequency ranges [36–39]. The concept of subjective alertness state was not addressed in these studies. Therefore, we do not know if qEEG correlates of sleepiness are present in stroke patients, in the same way as has been observed in nonbrain injured populations, nor to what degree this method can be used to measure sleepiness in this population.

In light of this gap in the literature, the present experiment therefore examined the qEEG in relation to subjective ratings of sleepiness in stroke patients with chronic hemiparesis. We hypothesised that the slowing of the daytime EEG, observed in the early stages of recovery, might be sustained in the chronic phase. Based on findings in healthy controls, we further predicted that increased power in the lower frequency bands is indicative of greater perceived sleepiness. Moreover, we hypothesised that these sleepiness parameters would be modulated by motor cognitive demand, and that this effect would be stronger in the presence of hemiparesis. Therefore, we applied a motor task in this study to facilitate motor cognitive demand.

## 2. Methods 

### 2.1. Design and Protocol

**Figure 1:**
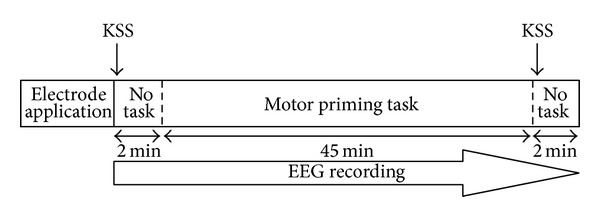
Study protocol.

This study employed a mixed within and between participant design. Each participant followed the protocol as outlined in Figure 1. After electrode application, waking EEGs were recorded for two minutes before and after the motor priming task with eyes open. During this time, participants were instructed to focus on a black dot in the centre of the screen as described in Karolinska Drowsiness Test [33]. A subjective sleepiness rating (Karolinska Sleepiness Scale; KSS) was recorded at the beginning of each two minute EEG recording at pre- and posttask (Figure 1).

### 2.2. Participants

Thirty-two community dwelling patients with first ever unilateral cortical or subcortical stroke >12 months were recruited via local general practitioner surgeries, hospitals, and online support communities. Average time since stroke was 69.1 months (±45.59, range 12–210). Eighteen patients had right, and 14 had left hemispheric strokes. Patients' mean age was 53.94 (±12.16 years, range 28–73) and 56% of the sample were male. All patients had approval from their general practitioner prior to taking part in the study. Exclusion criteria comprised a seizure within the past six months, severe balance difficulties, uncorrected visual impairment, uncontrolled comorbid illnesses or psychological disturbance, and diagnosed sleep disorders. The Mini Mental State Exam (MMS) [40] was applied in order to exclude those with cognitive deficits. The MME assesses orientation, immediate and short-term memory, attention, calculation, language, and spatial awareness. Total scores ≥25 are considered normal [40] and formed the exclusion criteria for this study.

The control group comprised twenty neurologically healthy participants recruited from the general population via flyers and posters. Exclusion criteria comprised presence of a brain injury or other serious health condition, diagnosed sleep disorder, and clinically significant psychological disturbance. Mean age was 54.1 years (±13.21, range 33 to 72) and 50% of the sample was male.

No participants were diagnosed with clinically significant sleepiness. The Epworth Sleepiness Scale (ESS) [23] was used as a measure of average sleepiness levels. ESS scores ≥10 are suggestive of significant problem with sleepiness. According to the ESS criteria, 28% of patients and 20% of controls reported significant sleepiness. Two out of five patients taking antiepileptic medication were above the ESS criteria. One out of four patients taking antidepressants was above ESS criteria. Mean ESS scores did not significantly differ between groups. Patients did not differ from controls apart from alcohol consumption where patients consumed significantly more than controls (*z* = −2.62, *P* < .01). Further analyses revealed no differences between patients with left or right hemispheric stroke. The demographics of the two groups are summarised in Table 1.

The study protocol was approved by the Surrey Research Ethics Committee (National Health Service UK) and the University of Surrey Research Ethics Committee. Written informed consent was obtained prior to participation. All procedures adhered to the ethical guidelines outlined by the Declaration of Helsinki [41].

### 2.3. Measures

Subjective sleepiness was measured with Karolinska Sleepiness Scale (KSS) [33], a one-dimensional scale to assess sleepiness at a particular time point. Scores ranged from 1 (very alert) to 9 (very sleepy). The KSS has shown good correlation with objective measures of alertness including EEG and vigilance tests [30], and in sleep deprivation paradigms [33, 42].

Waking EEG was used to record physiological indicators of sleepiness using a 64-channel QuickAmp system (Brain Products GmbH Munich, Germany) and Ag/AgCl electrodes using the standardised 10-10 montage [43]. Impedances were kept below 5 kOhm. Data was recorded in DC mode with a sampling rate of 500 Hz, and against average reference.

The motor task used Rosenberg's motor priming paradigm to facilitate motor cognitive demand in patients with chronic motor deficits poststroke [44].

One of four precues (left ≪, right ≫, either right or left <> or noresponse ><) were presented within an empty line drawn circle. Precues were immediately followed by one of three response cues represented by a black semicircle filled in white appearing within the line drawn circle: left half (left button press), right half (right button press), or bottom half circle (no response).

Participants were instructed to respond to the cues using the button press as quickly as possible. Precue presentations were randomised and were 100% predictive of the response cue. Following the response, a feedback screen was displayed for 500 ms and indicated either of the following: correct response (“correct”) or incorrect response (“wrong”; “not responded to response cue”; “a response was required!”) and responses within 200 ms of response cue (“too early!”). Sixty trials were presented approximately in 6 blocks. The response window was 1830 to 4000 milliseconds. Responses were executed with the left or right index finger, hand or arm, dependent on ability of patients who have some level of chronic hemiparesis. All participants first completed a training block to familiarise themselves with the procedure. Stimulus presentation was delivered using Neurobehavioural Systems Presentation Software (http://www.neurobs.com/).

### 2.4. Analysis

EEG signals were analysed offline using the Brain Analyzer Software (Brain Products GmbH Munich, Germany). A digital 0.5 Hz high pass and 30 Hz low pass phase shift-free Butterworth filter was applied as well as a 50 Hz notch filter. The two-minute segments of raw data were inspected manually for artefacts and further subdivided into two-second epochs. Frequency composition was determined through the Fast Fourier Transformation (FFT) module embedded in Visual Analyser. FFT criteria were set to full spectrum, resolution 0.5 Hz, power density output (*μ*V^2^/Hz), and a Hanning window (10%) was applied. The FFT values were averaged and exported in 0.5 Hz bins ranging from 1 to 30 Hz. These data were further subdivided into discrete, nonoverlapping frequency bands: delta (1–3 Hz), theta (4–7 Hz), alpha (8–12 Hz), and beta (13–30 Hz). Power density values were transformed using log base 10 (Log 10).

Differences in frequency composition between patients and controls were calculated for central electrodes (C3 and C4). Task and sleepiness effects were examined using central electrodes plus additional derivations including frontal (F3/F4), parietal (P3/P4), and occipital (O1/O2). Topographical distributions of discrete frequency bands were spline-interpolated [45] to create cortical maps for visual inspection.

To visualise group differences, geometric mean calculations were performed to express power density values of patients as a percentage of controls, a method previously used in nonbrain injured samples [46–48]. This was achieved by (1) transforming power density values using Log10, (2) averaging data across participants, (3) subtracting patient data from controls, (4) antilogging these values, and (5) multiplying by 100.

Performance on the motor task was measured as reaction time (ms), defined as time between response cue onset and button press, and the number of correct responses to the cue (%). For stroke patients, performance results were drawn from responses of the nonlesioned hand to minimise the impact of the motor deficit on performance. Reaction time and correct responses were averaged across blocks for all participants.

Statistical analysis was conducted using SPSS (SPSS Inc.; version 15.0). Between-group demographic variables were compared using *t*-tests or the Mann-Whitney *U* Test for nonnormal data. EEG data was log transformed using Log10. *T*-tests were used to directly compare the EEG of patients and controls for each 1 Hz bin between 1 and 30 Hz. Topographical maps of difference between patients and controls were plotted using the *P* values drawn from *t*-tests between groups per frequency band. Mixed-model ANOVAS with the repeated measures factors of task (pre-, post-) and hemisphere (left/right for controls; lesioned/nonlesioned for patients) and one between factor (group) were calculated to test for group and task effects on the KSS and EEG per frequency band. Significant findings were further examined using one-sided Fisher's protected *t*-tests with alpha set to *P* ≤ .05. For multiple comparisons, the Bonferroni correction was applied. Pearson's correlation coefficients were used to examine associations between KSS and EEG parameters. Correlations were Fisher's *z* transformed to allow the calculation of *Z*_obs_ score as described in Steiger [49]. *Z*_obs_ larger than 1.96 are statistically significant.

## 3. Results 

### 3.1. QEEG Characteristics in Patients versus Controls

**Figure 2:**
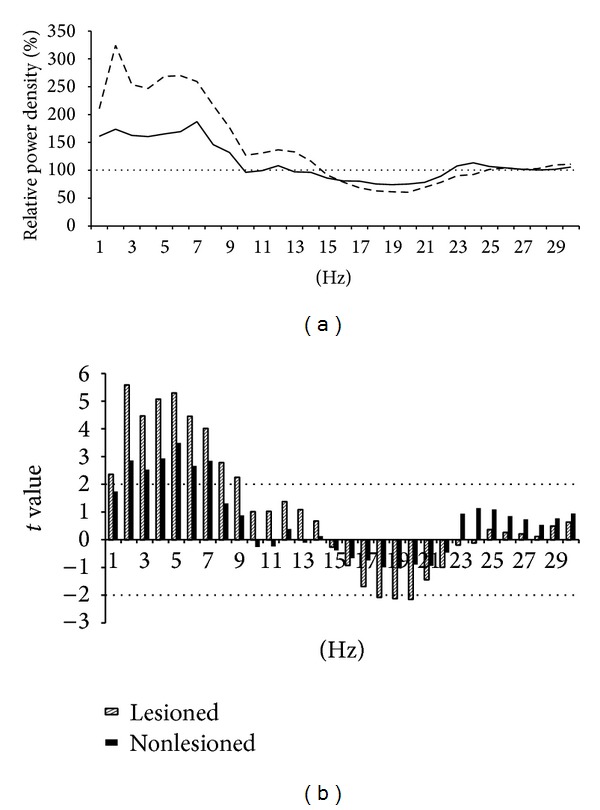
Difference in the EEG between patients and controls: (a) power density for the central derivation per 1 Hz for patients expressed as a percentage above or below controls at 100%; (b) between group *t* values with the dashed line to cut off point for significance.

Figure 2 presents the power density values where patient data are expressed as a percentage above or below control data. Patients showed greater power in the delta and theta ranges compared to controls (Figure 2), with significant increases between 1 and 9 Hz in the lesioned hemisphere and between 2 and 7 Hz in the nonlesioned hemisphere. At the same time, power in the higher frequency bands (18 to 20 Hz) was significantly decreased in the lesioned hemisphere. When correcting for multiple comparisons, this pattern remains the same whereby the cutoff for significance is a *t* value of 2.45, with the exception of increases at 1 and 9 Hz in the lesioned hemisphere.

**Figure 3:**
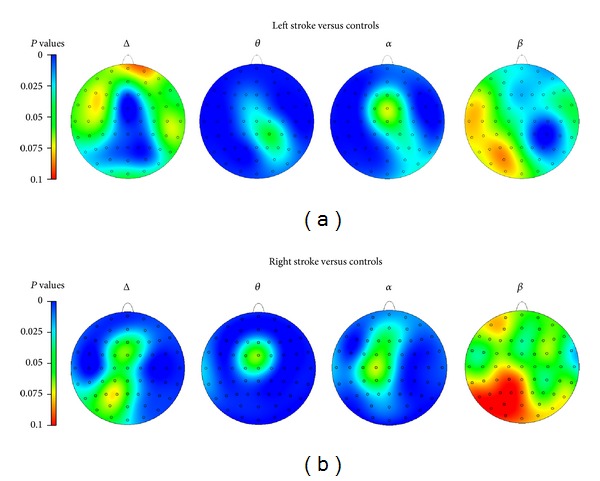
Topographical mapping of group differences in the EEG: level of significance for *t*-tests between (a) left (*n* = 14) and (b) right (*n* = 18) side stroke compared to controls is presented. When correcting for multiple comparisons of 64 electrodes, alpha level is *P* = 0.0008. This is indicated approximately by the grey line on the axes.

Topographic maps were calculated for patients with either left or right side stroke versus controls (Figure 3) by plotting the statistical difference between groups in the EEG power density for all electrodes (*P* value). Visual inspection shows a wide distribution of increased lower frequencies in both patients groups in comparison to controls. Left-lesioned patients show significantly greater frontal, central, parietal, and occipital slowing within the alpha and theta bands than controls. The right hemispheric group showed predominately frontal and parietal slowing in the delta band, as well as global slowing in the theta band, and increased alpha in frontal, parietal, and occipital regions compared to controls.

### 3.2. Effects of Motor Task on EEG and KSS

Reaction times in controls were significantly faster with a mean of 433 ms (SD = 67.70, range = 326–601; *z* = −3.24, *P* < .001) compared to 583 ms (SD = 239, range = 291–1664) in patients. The number of correct responses was significantly lower in patients (97.46%; SD = 5.17, range = 70–100) than controls (99.40%; SD = 0.72, range = 97.4–100; *z* = −3.20, *P* < .001).

**Figure 4:**
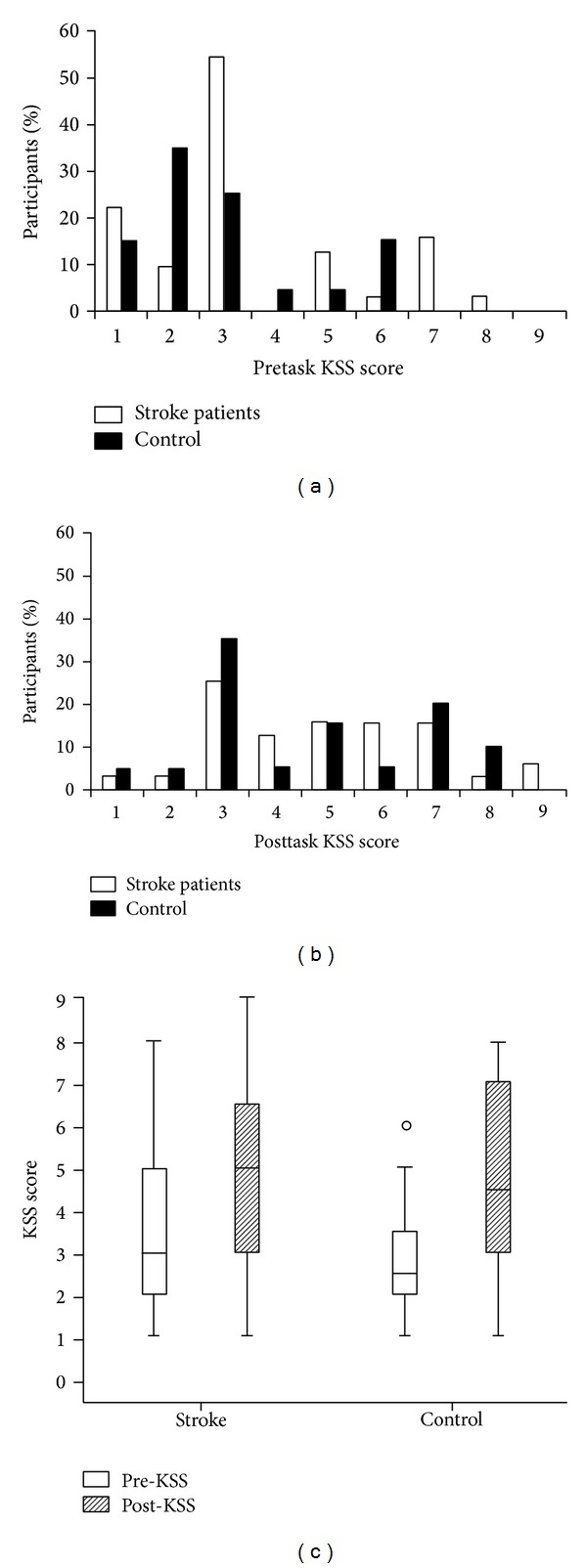
Changes in sleepiness ratings: (a) distribution of KSS scores pre-; (b) posttask; and (c) box plot displaying median KSS scores centrally in the box. Top and bottom values of the box represent the upper and lower interquartile range (H-spread) containing 50% of cases. The whiskers represent the highest and lowest scores which lie within 1.5 times the H-spread. Values >1.5 times the H-spread are outliers, represented by circles.

KSS scores are presented in Figure 4. Most participants scored ≤3, and the distributions for patients and controls do not indicate substantial differences. This is supported by the lack of significant group effect (*F*_1,50_ = 0.80, *P* = .37). A significant main effect task (*F*_1,50_ = 49.44, *P* ≤ .001) indicated that the subjectively perceived sleepiness was higher after completion of the task in all participants. Further inspection of KSS scores revealed a significant increase from 3.59 (±2.20) at pretask to 4.97 (±2.02) at posttask for patients (*t* = −4.63, *P* ≤ .001) and from 2.95 (±1.64) at pretask to 4.65 (±2.13) at posttask for controls (*t* = −6.03, *P* ≤ .001). The group differences at pre- and at post- were not significant.

Within-group task effects were computed separately for frontal, central, parietal, and occipital regions for each frequency band for lesioned and nonlesioned hemispheres in patients and controls (Table 2). Significant posttask increases in delta were revealed for patients in the frontal areas of the lesioned hemisphere. Increased theta was present in the lesioned hemisphere in both patients and controls. In patients, the nonlesioned hemisphere showed significantly increased theta only in the central region. For both groups, alpha power increased significantly with the task in all regions except for the parietal region in controls. In both groups, a significant posttask increase in alpha power was found across all regions apart from the parietal region of controls. Significant posttask increases within the beta band were observed for central and parietal regions in both patients and controls. In addition, controls did show an increase in frontal beta power at posttask. However, there were no changes in the beta band for frontal or occipital regions in patients. When correcting for multiple comparisons, the pattern remains the same when the alpha level is set to *P* ≤ .0.001 with the exception of changes from pre- to posttask in the parietal and occipital regions of controls.

The relative change from pre- to posttask in the EEG for all brain regions (frontal, central, parietal, and occipital) between lesioned and nonlesioned hemisphere within patients and controls was compared for each frequency band. A significant group difference was only revealed for alpha in the occipital area (*F*_2,81_ = 2.37; *P* < .05). Further analysis showed less occipital alpha in the lesioned hemisphere compared to controls (*t*_29_ = 1.87; *P* < .05). For the nonlesioned hemisphere, this effect showed a similar trend (*t*_50_ = 1.56; *P* = .06).

## 4. Associations

Correlations were computed for EEG power in each frequency band and the KSS. These correlations are listed for pre- and posttask in Tables 3(a) and 3(b).

At pretask, a significant association between increased KSS and increase central beta and occipital alpha was found for the lesioned hemisphere. For the nonlesioned hemisphere, higher pretask KSS was associated with greater central and parietal beta. In contrast, controls showed a significant association between higher KSS and increased global theta power as well as increased central alpha and higher KSS at pretask.

At posttask (Table 3(b), a relationship between occipital alpha and increased KSS was found for the lesioned he) misphere. In controls, increased posttask KSS was associated with increased theta in all derivations except occipital and increased alpha in the central and parietal areas. After correcting for multiple comparisons, the increase in occipital alpha for pretask patients remained significant as did the frontal and central theta in controls.

Post hoc analysis using Fisher's *z* transformation revealed that the only difference between controls' and patients' correlation coefficients that approached statistical significance was that for the theta band (*Z*_obs_ ranged between 1.31 and 1.71).

## 5. Discussion

The present study used EEG recordings to examine whether patients in the chronic state show alterations in their wake EEG and to investigate how these changes are related to perceived sleepiness. We further sought to determine whether motor cognitive demand would modulate the wake EEG in patients and controls. Overall, the results confirm our initial hypothesis that the prevalence of lower frequencies in the wake EEG, characteristic of the acute and subacute phase of stroke, is sustained in the chronic phase. However, in contrast to our expectations, this slowing of the EEG was not associated with an increase in perceived sleepiness in patients. In other words, the EEG characteristics, commonly accepted as biomarkers for sleepiness in healthy persons, are present in the wake EEG of patients with chronic stroke but do not seem to be associated with greater perceived sleepiness.

To the best of our knowledge, this is the first study using a qEEG-based approach to examine sleepiness in a chronic stroke population with sustained motor deficits. The various findings and their implications are discussed below.

### 5.1. Wake EEG Characteristics in Patients

Compared to controls, an increase in slow (≤9 Hz) frequencies was observed in both hemispheres. This slowing covered large areas of the cortex including frontal, parietal, and occipital areas as indicated by the topographical maps. The right stroke group showed more delta, particularly in frontal and central regions. These findings are in line with reports of increased global delta and theta during the acute phase of stroke [36, 38, 39]. The only study which examined qEEG in chronic stroke patients beyond one year used a single electrode in the frontal area and showed significantly more delta in patients [50]. The present findings therefore expand existing knowledge on the characteristics of the wake EEG in the chronic state. The data suggests that changes in EEG frequency, in particular the lower frequency bands, are sustained in the chronic phase of stroke and that these changes affect both the lesioned and the nonlesioned hemispheres. Interestingly, significant differences between the hemispheres were observed for frequencies >11 Hz, with greater beta power over the nonlesioned hemisphere, which may reflect a compensatory mechanism. No hemispheric differences were found for controls. In addition to the increased prevalence of low frequencies, earlier stages of recovery are marked by hemispheric dissymmetry [38, 51–53]. The present findings therefore suggest that the wake EEG in the chronic state maintains some key characteristics of the acute phase, namely, a greater prevalence of lower frequencies and a hemispheric dissymmetry in the beta range.

### 5.2. Effects of the Motor Task

Both patients and controls reported posttask increases in the KSS, suggesting greater sleepiness after the task. Posttask changes were also observed in the EEG, with both groups showing predominantly increased alpha and theta. Theta increases were consistent between the lesioned hemisphere and controls in frontal, central, and occipital regions. In the nonlesioned hemisphere, only the central area reached significance for increased theta. Critically, patients also showed increased frontal delta in the lesioned hemisphere which was not observed in the nonelesioned hemisphere or for controls. In other studies with healthy participants, posttask increases in the alpha range are indicative of mild subjective sleepiness, whereas increased delta activity is suggestive of more severe sleepiness [54, 55].

With regard to between-group effects, there was significantly less posttask change in occipital alpha in the lesioned hemisphere compared to controls and the nonlesioned hemisphere, while controls and the nonlesioned hemisphere did not differ. In this sense, the nonlesioned hemisphere “responded” to the task in a similar fashion as controls, while the lesioned hemisphere responded differently. This suggests that the hemispheric dissymmetry might not just be a physiological epiphenomenon but a characteristic that is functionally important for cognition and behaviour.

In addition to changes in the lower frequency bands, we further observed a posttask increase in beta in patients, in both hemispheres, and controls. Notably, this increase in higher frequencies occurred at the same time as an increase in lower frequencies, an effect also described in studies requiring sustained mental effort in control populations [56, 57]. This effect most likely reflects that the compensatory effort participants have to make in the face of declining vigilance as the time-on-task increases [56]. However, this theory has not been tested directly in chronic stroke patients.

### 5.3. Associations

The present study has found that subjective sleepiness ratings strongly correlated with increased pre- and posttask theta and alpha in controls, corroborating previous work in this area [33–35]. The finding is in line with the generally accepted assumption that the EEG-derived neurophysiological markers of sleepiness are directly related to the subjective perception of sleepiness and indeed reflect greater sleep propensity [33]. In patients, the relationship between subjective sleepiness and EEG indices was much less prominent, with only near significant correlation coefficients within the theta band. This dissociation between subjective and objective sleepiness markers is an interesting finding and clearly requires further investigation. One possible explanation is that patients habituate to their chronic sleepiness over time and therefore fail to perceive or report it. In this context it is interesting to note that other studies have found poor consistency between self-reported sleepiness and other indicators of sleepiness in patients, including observable report, actigraphy, and indeed the MSLT. Moreover, Sforza et al. [58] found no correlation between EEG and self-reports in patients with sleep disorders and concluded that the wake EEG is not sensitive to sleepiness in this population. The same argument may be put forward for the present study. However, our own observations during the experiment indicated that patients were indeed rather sleepy. This was shown in patients' behavioural characteristics of sleepiness including yawning, eyes closed, and a nodding head, while the electrodes were attached. The subjective sleepiness ratings therefore stand in contrast to the behavioural signs of sleepiness we observed. We therefore conclude that it is likely that patients have greater difficulty with the perception of their sleepiness, a hypothesis which could be explored using the MSLT. In addition, a larger sample size would build on the results from this study.

Increased beta weakly correlated with higher KSS in patients at pretask only and this effect was not observed in controls. This further provides more direct support to the compensation theory as discussed above. We hypothesise that the increased beta is indicative of the allocation of cognitive and behavioural resources, necessary to compensate for the limited attentional capacity [10]. This may signify a sustained compensatory beta enhancement that is necessary for patients to function in everyday life. At posttask, this effect disappears and may reflect an exhaustion of maximum attention capacity.

### 5.4. Limitations and Further Theoretical Considerations

Even though the results show significantly more slowing of the EEG in patients compared to controls, which is similar to that shown in sleep deprived persons, the cause of sleepiness cannot be identified. It may be the result of several factors including disruption to alertness mechanisms as direct result of lesion location [16], poor sleep [21], low mood [17], and increased effort required in movement and cognition after stroke [22]. It is likely that sleepiness may be the combined effect of several factors. Regardless of cause, the results show that patients are poor at recognising alertness state even though there is clear evidence of EEG slowing in both hemispheres, similar to that seen after sleep deprivation.

The participant sample has some points for consideration. Due to the sample size, the statistical power is small and therefore more studies are necessary to further explore measuring sleepiness in stroke populations. The sample included participants taking medication that may affect the EEG including antiepileptics [59] and antidepressants [60, 61]. Although there are other factors contributing to increased sleepiness as described in the introduction, we cannot decipher the degree to which these factors interact with the EEG and perception of sleepiness. However, had medication significantly affected the patients' EEG, we would expect the effect to be observed in both hemispheres. Furthermore, some antidepressants may even increase vigilance [61]. Overall, we aimed to capture a representative sample of stroke patients which would typically include those who engage in behaviour that can contribute to changes in physiological arousal such as medication usage but are not directly related to their stroke per se. We have shown that regardless of cause of sleepiness, the observed EEG slowing is potentially problematic in patient prognoses after stroke and requires attention.

## 6. Conclusion 

The present study shows long-term changes of the wake EEG in patients with chronic hemiparesis after stroke. The data suggests a general slowing of the EEG that affects the lesioned and nonlesioned hemisphere. This slowing is not associated with subjective sleepiness and this has implications for the recognition and treatment of an aspect of stroke that impacts rehabilitation and safety. Investigating the causes of this dissociation will add to a better understanding of the long-term consequences of stroke and eventually help to improve stroke care.

## Figures and Tables

**Table 1:** Participant information: data presented as mean, ± 1 standard deviation, range, or percent.

Demographical variables	Stroke patients (*n* = 32)	Controls (*n* = 20)
Gender (M : F)	18 : 14	10 : 10
Age (Years)	53.97 ± 12.16 (28–73)	54.10 ± 13.21 (33–72)
BMI	24.10 ± 2.56 (18.20–28.90)	24.56 ± 3.64 (18–30.80)
MMSE	29.10 ± 1.06 (26–30)	—
ESS	6.69 ± 4.41 (0–17)	5.45 ± 4.52 (0–15)
Chronicity	60.91 ± 45.59 (12–210)	n/a
Stroke hemisphere (Left : Right)	14 : 18	n/a
Medication (frequency of participants on medications)	Antidepressant (4) Cardiac control (4) Antiepileptic (5) Sleep hypnotics (1)	n/a
Alcohol (units per week)	8.98 ± 10.75 (0–45)	3.66 ± 8.12 (0–35)
Caffeine (servings per day)	4.36 ± 2.73 (0–12)	3.31 ± 2.74 (0–10)
Nicotine (cigarettes per day)	0.97 ± 3.90 (0–20)	0.50 ± 2.24 (0–10)

**Table 2:** Pre- to Posttask change in EEG power (*t* values): negative values indicate an increase in EEG power. Significant changes are identified as * ≤0.05, ** ≤0.001, and *** ≤0.0001.

	Delta	Theta	Alpha	Beta
Lesioned hemisphere				
Frontal	−2.84**	−3.42**	−3.60***	−1.86
Central	−0.73	−2.73**	−3.54**	−3.69**
Parietal	−0.40	−1.58	−2.50**	−4.39***
Occipital	−0.93	−2.70**	−2.99**	−0.41
Nonlesioned hemisphere				
Frontal	−0.57	−1.91	−3.15**	−1.42
Central	−0.64	−2.34*	−5.38***	−3.68***
Parietal	−1.62	−1.28	−3.57**	−4.72***
Occipital	−1.60	−1.82	−3.38**	−0.61
Controls				
Frontal	−0.70	−4.06***	−4.32***	−4.96***
Central	0.79	1.79	−3.38**	−4.63***
Parietal	−0.32	−2.11*	−1.90	−2.72**
Occipital	−1.42	−2.31*	−3.67**	−1.01

**Table:** (a)

Pretask	Delta	Theta	Alpha	Beta
Lesioned hemisphere				
Frontal	0.04	0.12	0.20	0.24
Central	0.10	0.09	0.15	0.31*
Parietal	0.11	−0.03	0.13	0.18
Occipital	0.15	0.07	0.39**	0.16
Nonlesioned hemisphere				
Frontal	0.07	0.02	0.18	0.16
Central	0.07	0.14	0.24	0.31*
Parietal	−0.02	0.06	0.15	0.35*
Occipital	0.07	0.06	0.24	0.19
Control				
Frontal	0.23	0.51**	0.31	0.04
Central	0.28	0.56**	0.44*	0.33
Parietal	0.34	0.43*	0.24	0.19
Occipital	0.14	0.44*	0.14	0.26

**Table:** (b)

Posttask	Delta	Theta	Alpha	Beta
Lesioned hemisphere				
Frontal	0.20	0.18	0.30	0.05
Central	0.15	0.04	0.19	0.13
Parietal	0.05	−0.14	0.05	−0.05
Occipital	0.17	0.10	0.36*	0.10
Nonlesioned hemisphere				
Frontal	0.13	0.06	0.21	−0.12
Central	0.26	0.18	0.34*	0.09
Parietal	0.09	0.16	0.31*	0.06
Occipital	0.17	0.20	0.32	0.13
Control				
Frontal	0.35	0.39*	0.24	−0.03
Central	0.05	0.37*	0.48*	0.41
Parietal	0.25	0.41*	0.38*	0.33
Occipital	−0.11	0.27	0.09	0.19
